# Uncertainty-aware physics-informed meta-learning for predicting niosomal particle size across formulations and processing conditions

**DOI:** 10.3389/fmed.2026.1966154

**Published:** 2026-09-30

**Authors:** Ahmad J. Obaidullah, Wael A. Mahdi, Adel Alhowyan

**Affiliations:** 1 Department of Pharmaceutical Chemistry, College of Pharmacy, King Saud University, Riyadh, Saudi Arabia; 2 Department of Pharmaceutics, College of Pharmacy, King Saud University, Riyadh, Saudi Arabia

**Keywords:** machine learning, niosomal formulations, particle size prediction, prediction interval coverage probability, uncertainty quantification

## Abstract

Accurate prediction of niosomal particle size across diverse formulation compositions and processing conditions remains challenging because experimental datasets are typically limited and heterogeneous. This study presents an uncertainty-aware physics-guided meta-learning (PGML) framework for predicting the mean particle size of niosomal formulations prepared using the thin-film hydration (TFH) method. The framework integrates molecular descriptors, formulation variables, and processing conditions within a shared meta-learning representation, while soft formulation-guided regularization is used to discourage physically implausible model behavior. A probabilistic Gaussian-process prediction component provides predictive uncertainty together with particle-size estimates. The framework was evaluated using 114 formulations collected from 17 published studies and an independent test partition. The final model achieved an RMSE of approximately 0.65, an MAE of 0.43, and an R^2^ of approximately 0.90, while the 95% prediction interval coverage probability (PICP@95%) was approximately 0.90. The results indicate that combining soft physics-guided regularization, task-based learning, and probabilistic prediction can provide accurate particle-size estimates together with information regarding predictive confidence. The proposed framework is intended as a formulation-level decision-support approach for screening and prioritizing experimental conditions when available data are limited and heterogeneous.

## Graphical Abstract

**Figure:**
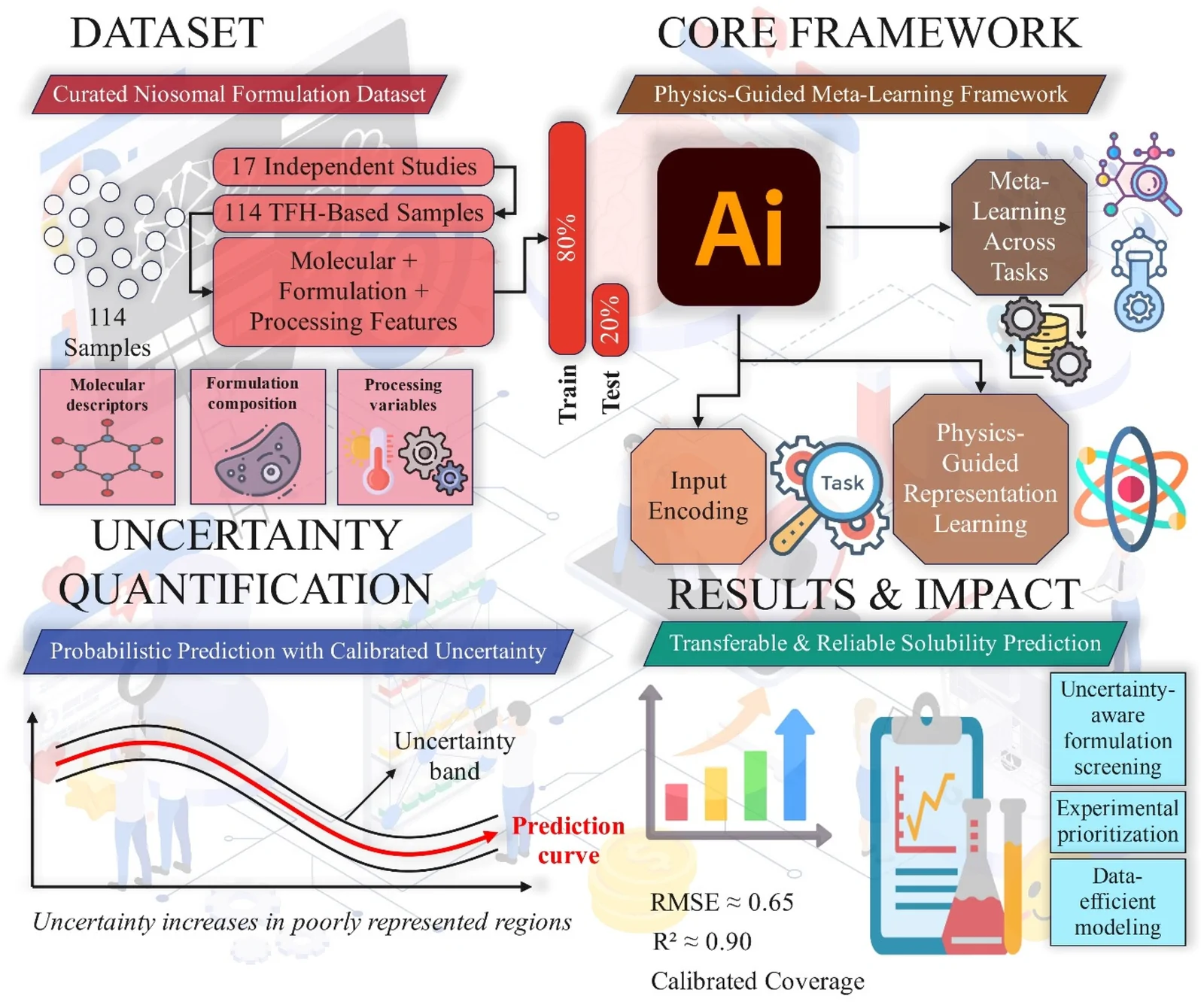

## 1 Introduction

Niosomes are non-ionic surfactant-based vesicular systems composed primarily of non-ionic surfactants and membrane-stabilizing components such as cholesterol. Their ability to accommodate hydrophilic and hydrophobic drug molecules, together with their formulation flexibility and physicochemical stability, has made them important carriers in pharmaceutical and nanomedicine research. The physicochemical properties of niosomes (1, 2), particularly particle size, size distribution, membrane characteristics, encapsulation efficiency, and stability, are strongly dependent on both formulation composition and preparation conditions. Among these properties, particle size represents a particularly important critical quality attribute because it influences vesicle surface area, physical stability, drug release behavior, cellular interactions, and the suitability of a formulation for specific delivery routes (3). Particle size is not governed by a single formulation variable (4). Instead, it emerges from coupled interactions among drug physicochemical properties, surfactant characteristics, lipid composition, formulation ratios, and processing conditions. In thin-film hydration (TFH), for example, the hydration temperature, hydration time, solvent conditions, and composition of the lipid/surfactant film collectively influence bilayer formation and vesicle assembly. Recent reviews have emphasized the importance of these preparation variables for controlling niosomal particle size and structural properties (5–7).

The hydrophilic–lipophilic balance (HLB) of the surfactant is particularly relevant because it influences surfactant hydration, interfacial behavior, and vesicle formation. Experimental evidence has indicated that changes in HLB can substantially affect niosomal particle size. Similarly, cholesterol acts as a membrane-stabilizing component and modifies bilayer rigidity, permeability, and vesicle stability. The effect of cholesterol on particle size can depend on the surfactant system, indicating that formulation variables may exhibit nonlinear and interacting effects rather than simple universal relationships (8, 9). Fragment-based semi-empirical approaches, such as the general solubility equation, UNIFAC, UNIQUAC, and PC-SAFT, are examples of traditional solubility prediction techniques. These approaches have limits because of their simplifications and presumptions, which present challenges, particularly when dealing with a variety of chemical compounds (10). Furthermore, some approaches' reliance on empirical parameters limits their generalizability by making it difficult to apply them to novel substances beyond the purview of the experimental data. However, through in-depth simulations and electronic structure calculations, approaches based on molecular dynamics, and first principle *ab initio* calculations (such COSMO-RS) provide insights into molecular behavior (11). Because of these restrictions, there is an increasing interest in data-driven modelling approaches that can learn complex solubility relations directly from experiments (12). Such methods have the possibility of capturing the nonlinear interactions between molecular properties, formulation variables, and processing conditions, while depriving the dependence on restrictive assumptions or heavy parameterization, that are the features of a traditional physics-based methods (13) (Figure 1).

**Figure 1:**
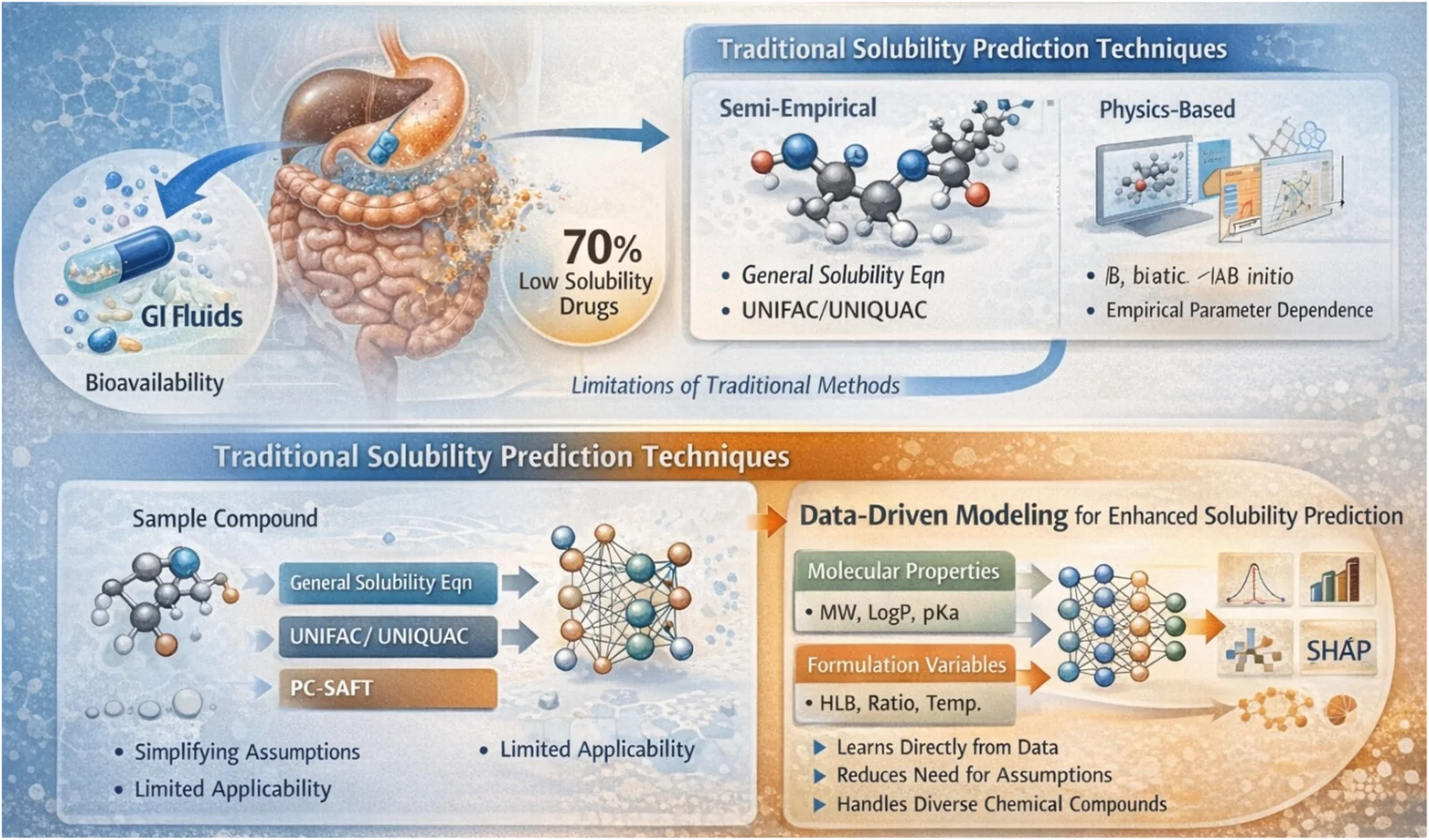
Schematic overview of the challenges of poor drug solubility and the progression from traditional solubility prediction methods to modern data-driven modeling approaches in pharmaceutical development.

In recent years, ML techniques have been improved to such an extent that the prediction of drug solubility can be successfully carried out for a wide range of chemically diverse compounds and different formulation conditions. For instance, Bao and his colleagues created several ML models which were trained on a very large dataset containing nearly 27,000 data points, the aim of the models was to forecast drug solubility in different binary solvent mixtures over a very wide temperature range (14). Their work observed that drug solubility in mixtures in the main domain of the training set was very accurately predicted and that the factors of solvent composition and temperature changes must be explicitly accounted for. The availability of open datasets and tools further supports reproducibility and future methodological development. Some research has unfolded the impact of molecular representation when it comes to predicting aqueous solubility. A comparative report released in 2023 scrutinized descriptor-based and fingerprint-based ML models with a dataset of about 8,400 organic compounds; it was found that feature representation plays a key role in the accuracy and stability of models (15). Further developing this work, Ghanavati, Ahmadi, and Rohani made a very detailed assessment of the various ML structures, including graph-based, tabular, and potential-map representations, checked against four benchmark solubility datasets (16). The study pointed out that not only the proper choice of the model but also the input representation significantly influences the predictive power and the ability of a model to generalize cross-datasets. In their work, Zhao et al. suggested a multi-task graph transformer model that could predict intrinsic solubility concurrently with other physicochemical properties (17). They trained their model on a dataset of over 13,000 drug-like molecules. Their multi-task model consistently beat single-task models and random forest baselines, showing the benefits of shared representation learning. Likewise, Wang et al. used Hansen solubility parameters as additional features to ML models for the prediction of solubility in complex micro-environments (18). Their results demonstrated that adding mechanism-informed descriptors to prediction models can drastically increase the precision of solubility prediction, especially in the cases of heterogeneous solvation systems. Ngwu has given a broader perspective to the field by reviewing the most recent ML models developed for the prediction of aqueous solubility in the area of drug discovery, ranging from random forests, support vector machines to deep learning techniques (19). According to this review, the challenges that have been constantly faced include poor quality of data, lack of uncertainty quantification, and no systematic approach to interpretability. Using ML to tackle the problem of solubility prediction in realistic processing conditions, Lahiq et al. adapted the ML models to be used in continuous manufacturing environments with green solvents and thus, demonstrated that the models are capable of handling variations in temperature and pressure (20).

Reliable estimates of the average particle size in the TFH-prepared niosomal formulations are still not achieved. The problem of predicting models when formulation compositions and/or process conditions are different from the one present in the training data is still a big challenge. This paper, in response to the demand for prediction frameworks that are robust and transferable to various niosomal systems rather than narrow task-specific optimization, introduces the possibility of predicting the mean particle size of the niosomal formulations prepared by thin-film hydration under varying formulation and processing conditions by jointly considering compositional variables and experimentally controllable manufacturing parameters. One of the main objectives of this work is to make sure that the prediction results remain consistent with the principles of physicochemistry and process engineering, which minimizes the chances of getting unrealistic or unreasonable estimates when one extends the predictions to new formulation spaces. This paper, however, does not only pay attention to predictive accuracy, but also incorporates the issue quantification of uncertainties that formulation decision-making could rely upon. The framework is expected to be an aid for doing risk-based evaluation of formulation options, selecting key experiments for testing, and optimizing production processes by providing uncertainty estimates together with particle size predictions. All these aims are meant to push the boundary in the development of transferable, physically consistent, and decision-orienting predictive models for niosomal formulation design and pharmaceutical development.

## 2 Dataset description and curation

The dataset in this study derives from published results of Shahiwala et al. (21), who had previously compiled various data from 17 independent peer-reviewed articles which discussed niosomal drug formulation prepared through the thin-film hydration method. The literature review in this prior work involved a systematic approach of PubMed and Google Scholar databases with well-structured, pre-defined keywords focusing on niosomes, Span 60, and TFH-based formulation. The authors followed the PRISMA framework throughout the study to keep the screening method open to interpretation and ensure high-quality data. Only those papers which were detailed enough to extract full molecular descriptor values, formulation variable details, processing condition data, and experimentally-measured particle sizes were considered. Those papers which were missing quantitative data or used completely different preparation techniques weren't included.

The dataset finally generated consisted of 114 different niosomal formulations that were used in various drug delivery applications of diverse drugs, formula compositions, and preparation conditions. Being a collection of data taken from various laboratories and measurement systems and procedures, this database shows quite a bit of experimental variation in formulation work. The learning system was not expected to eliminate this variance but on the contrary, such heterogeneity being a matter of fact had helped to test that the framework could still be capable of establishing relationships between various formulations without much interference by differences caused by different experimental settings. In order to use the dataset at hand for the current project, the authors performed modifications that would suit a supervised machine learning scenario. Specifically, molecular descriptors, drug formulation composition, and processing variables were included as input features. Meanwhile, the mean particle size (nm) of the obtained niosomes in formulations was used as the output. The full list of references of the original studies has also been provided at the References part of this paper and is marked as (21–38), following the original dataset compilation work of Shahiwala et al.

### 2.1 Input features and target output

Each experimental sample is represented using a structured set of 11 input features describing the physicochemical characteristics of the drug, formulation composition, and TFH processing conditions. These variables constitute the complete input feature space of the predictive model. The target output is the experimentally measured mean particle size of the resulting niosomal formulation (nm). The input variables include molecular weight, lipophilicity (LogP), ionization constant (pKa), intrinsic solubility, hydration medium amount, solvent amount, hydrophilic–lipophilic balance (HLB), drug-to-lipid ratio, cholesterol-to-Span 60 ratio, hydration temperature, and hydration time. Particle size is not supplied to the model as an input feature at any stage of training, adaptation, hyperparameter optimization, or testing. This distinction is important because particle size represents the response variable that the framework is designed to predict. Accordingly, no particle-size-derived variable is used as a predictor, thereby preventing target leakage. Table 1 summarizes all input features and the output variable, together with their physical meaning and functional roles within the modeling framework.

**Table 1:** Summary of model input features and target output variables.

Category	Feature	Symbol	Unit	Description	Functional role
Molecular descriptors	Molecular weight	MW	g·mol⁻¹	Mass of drug molecule	Controls diffusion, partitioning, and dissolution kinetics
	Lipophilicity	LogP	–	Octanol–water partition coefficient	Governs membrane affinity and vesicle encapsulation
	Ionization constant	pKa	–	Acid–base dissociation constant	Determines ionization state and solubility behavior
	Intrinsic solubility	$S_{0}$	mg·mL⁻¹	Solubility of neutral form in water	Baseline solubility reference
Formulation parameters	Cholesterol–surfactant ratio	Chol/Span	–	Cholesterol to Span 60 mass ratio	Controls membrane rigidity and vesicle stability
	Drug-to-lipid ratio	D/L	–	Drug loading fraction	Regulates encapsulation efficiency and release behavior
	Hydrophilic–lipophilic balance	HLB	–	Surfactant polarity index	Affects vesicle formation and interfacial stability
	Solvent volume	V_sol	mL	Organic solvent amount	Influences lipid film formation and hydration efficiency
Processing conditions	Hydration temperature	T_hyd	°C	Film hydration temperature	Controls lipid fluidity and vesicle assembly
	Hydration time	t_hyd	min	Duration of hydration step	Affects vesicle maturation and drug incorporation
Target output	Mean Particle size	D_p	nm	Experimentally measured mean vesicle diameter	Prediction target

### 2.2 Dataset characteristics and partitioning

The dataset encompasses 114 niosomal formulations collected from 17 independent eligible studies. The original literature-based dataset was constructed by screening studies using predefined criteria and extracting formulations for which the required molecular, formulation, processing, and particle-size variables were available. For the meta-learning procedure, the 17 source-study-derived formulation families provided the task-level structure. Formulations originating from the same formulation family were treated as observations within a common task because they shared a related experimental and formulation context. During meta-training, model performance was monitored using held-out validation tasks to assess the evolution of predictive performance across formulation families not used for the corresponding meta-level updates. This task-level validation was used to monitor the meta-training process, whereas the final predictive evaluation was conducted using the independent test partition consisting of 22 formulations. Thus, task-based validation during meta-training and the final sample-level test evaluation served distinct purposes: the former monitored learning across formulation tasks, while the latter provided the final independent assessment of predictive and uncertainty performance.

For the final sample-level evaluation, the dataset was partitioned into 92 training samples and 22 independent test samples, corresponding to approximately 80.7% and 19.3%**,** respectively. This represents the intended approximately 80/20 partition while maintaining integer sample counts for the 114 observations. The 22 test samples were kept independent from model fitting and were not used for hyperparameter selection or final model optimization. To reduce information leakage, observations belonging to the same formulation family were assigned consistently to the same partition wherever applicable. Consequently, closely related formulations from a common experimental family were not deliberately distributed between training and testing sets.

### 2.3 Experimental procedure

This research mainly uses experimental data from articles in which authors have prepared niosomal formulations with the TFH method, a very common method for vesicular drug delivery systems (21). Firstly, in their experimental work, non-ionic surfactants (most commonly Span 60) together with cholesterol were dissolved along with the drug in an organic solvent mixture to obtain a homogeneous lipid phase. Afterwards, the solvent was removed under reduced pressure by rotary evaporation, which led to a thin lipid film being deposited on the flask wall. The film was kept in the dry state and then hydrated with a suitable aqueous medium at a temperature controlled by agitation, which resulted in the spontaneous generation of multilamellar niosomal vesicles. Researchers changed hydration time, temperature, and volume of aqueous phase to control vesicle formation and drug entrapment efficiency. Some post-hydration processing steps like sonication or size reduction have been adopted by the researchers to get narrower particle size distributions, which are more suitable for characterization. Later, physicochemical standard techniques were used for the characterization of the formulations. In fact, dynamic light scattering is the method that authors prefer for the measurement of particle size and particle size distribution, whereas encapsulation and solubility-related features are determined after centrifugation or filtration methods separate free drug from vesicle-bound drug. The drug concentration in the aqueous phase is often determined with spectrophotometric or chromatographic techniques to get the drugs' solubility and formulation performance values. The dataset that has been put together thus consists of the results of experimental measurements made under highly controlled preparation and characterization conditions, with formulation composition and processing parameters systematically varied so that their effect on drug solubility and vesicle properties could be induced.

### 2.4 Data preprocessing and analysis readiness

Figure 2 illustrates the uncertainty landscape of predicted niosomal particle size across the formulation–process parameter space. The contour heatmaps visualize the magnitude of predictive uncertainty across combinations of molecular, formulation, and processing variables. Higher uncertainty is observed in regions characterized by extreme drug-to-lipid ratios, lower solvent amounts, and shorter hydration times, where the available experimental data provide relatively limited coverage. In contrast, lower uncertainty is observed across intermediate regions of the formulation and processing space that are more densely represented in the dataset. The smooth variation in uncertainty across neighboring conditions indicates structured changes in predictive confidence rather than abrupt fluctuations. These patterns suggest that the uncertainty estimates provide useful information regarding regions in which additional experimental observations may be particularly valuable.

**Figure 2:**
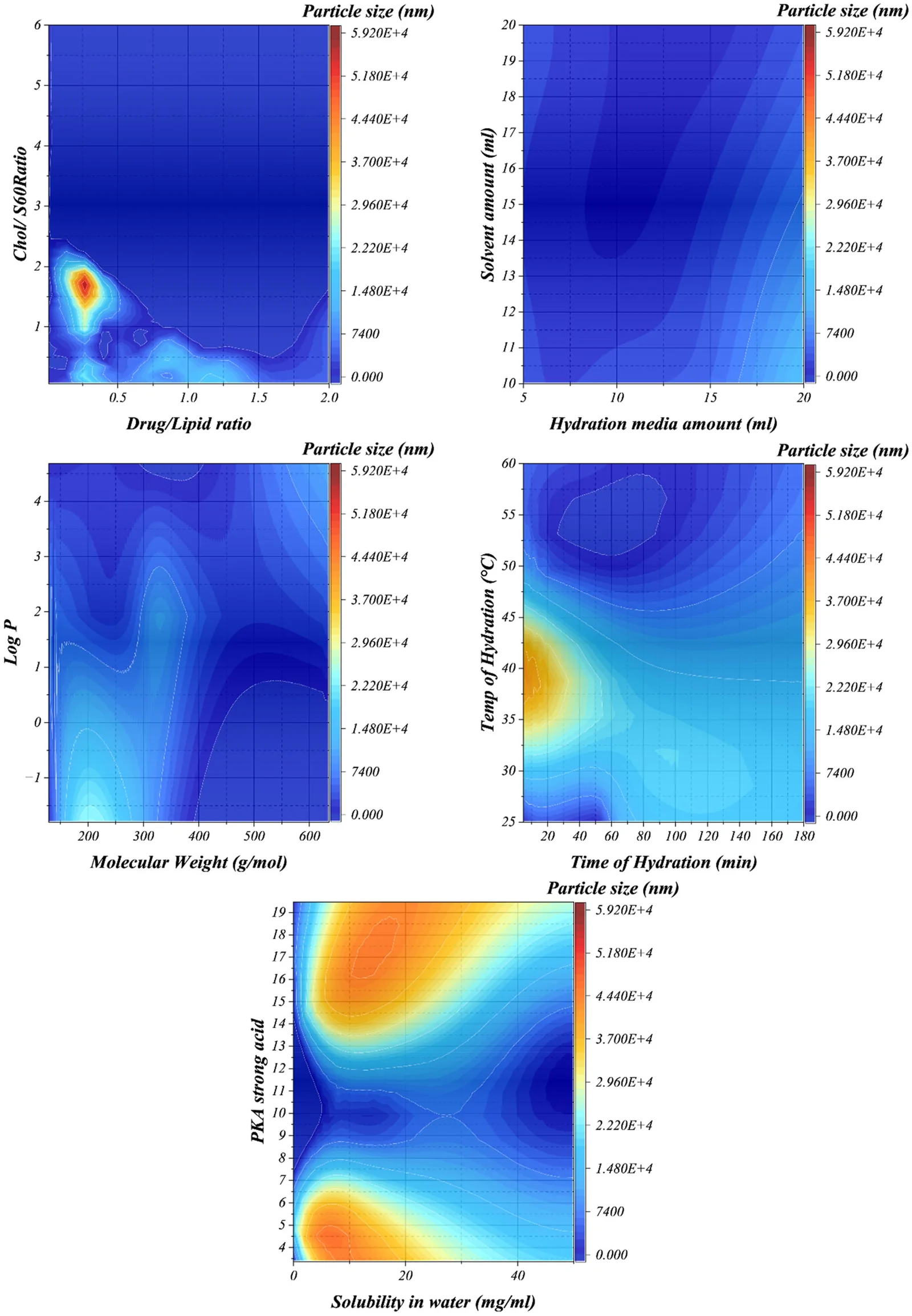
Uncertainty landscape of predicted niosomal particle size across the formulation–process parameter space. The contour heatmaps visualize the magnitude of predictive uncertainty across combinations of molecular, formulation, and processing variables, with higher uncertainty indicating regions of lower predictive confidence.

Figure 3 shows the pairwise correlation heatmap of molecular, formulation, and processing variables that were integrated into the physics-based meta-learning framework. The overall correlation matrix suggests that there are mainly weak to moderate correlations among most of the variable pairs, which indicates there are no strong multicollinearities present in the input space. In other words, the predictors have limited linear redundancies between them and the feature set is well-conditioned for multivariate modeling. The stronger correlations seem to be limited to chemically intuitive relationships, for instance, the correlation between molecular weight and LogP as well as the negative correlation between intrinsic solubility in water and hydrophobicity related descriptors.

**Figure 3:**
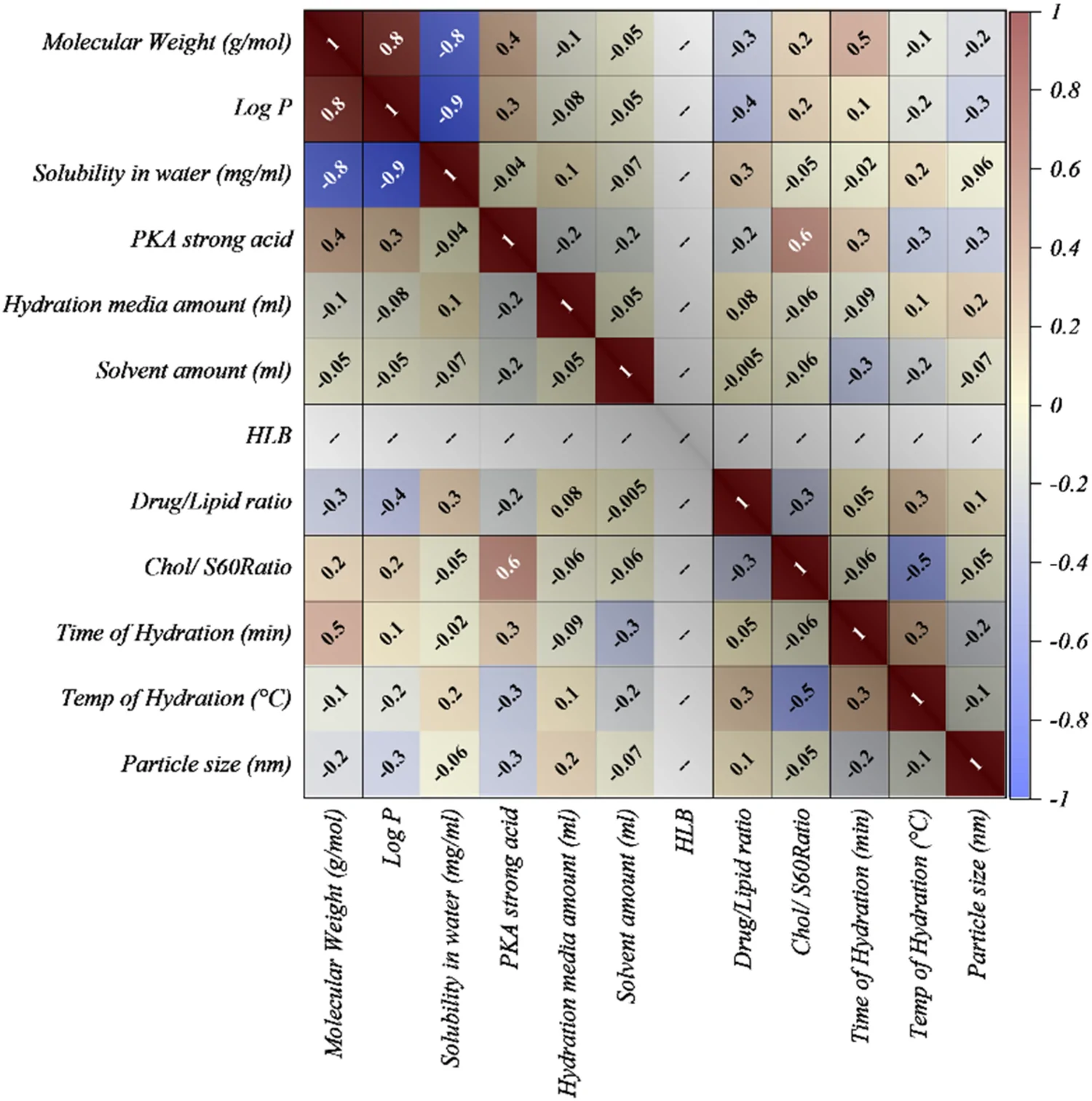
*Correlation structure of molecular, formulation, and processing variables used in the physics-guided meta-learning model*.

## 3 Methodology

The modeling framework aims to solve the problem of predicting particle size for diverse molecular structures, compositions of formulations, and processing conditions, especially when experimental data are limited and extrapolation is necessary. Input variables comprise molecular descriptors (molecular weight, lipophilicity, and ionization constant) together with formulation and processing parameters such as hydrophilic–lipophilic balance, drug-to-lipid ratio, solvent amount, hydration time and temperature, and particle size. The selection of these inputs was based on experimentally controllable factors that are typically reported in formulation studies and the need to learn from varied formulation regimes. A multi-layer neural network serves as a feature encoder to convert heterogeneous inputs into a shared latent representation (39). This shared representation was chosen because the particle size behavior is the result of coupled interactions between molecular properties and formulation or processing variables, and thus, particle size expression is not separable by isolated descriptors. The latent space embedding allows the model to capture drug- and formulation-family-dependent transferable structure, while still being sufficiently flexible to adapt to the new experimental settings.

### 3.1 Physics-guided learning

The predictive model incorporates formulation knowledge through a soft physics-guided regularization term. The purpose of this term is not to impose a deterministic mechanistic equation for niosome formation, but to discourage predictions that are inconsistent with established formulation behavior while retaining sufficient 'exibility to learn nonlinear interactions from the experimental data (39). Equations 1–10 present the basic mathematical formulas of the methods used in this study. The total objective function is defined as

$$L=L_{\mathrm{pred}}+\lambda L_{\mathrm{physics}}$$ (1)

where $L_{\mathrm{pred}}$ is the prediction loss, $L_{\mathrm{physics}}$ represents the formulation-guided penalty, and λ controls the contribution of physical regularization. In the final model, the physics weight was set to 0.2, as reported in Figure 7. The principal physical priors considered in the regularization are summarized below.

#### 3.1.1 HLB-related constraint

The HLB of a nonionic surfactant influences its hydrophilic/hydrophobic balance and therefore its hydration and vesicle-formation behavior. Literature evidence indicates that increases in HLB can be associated with increases in niosomal particle size in relevant surfactant systems. Accordingly, a soft monotonicity penalty can be expressed as

$$P_{\mathrm{HLB}}={\left[\mathrm{ReLU}\left(-\frac{\partial {\hat{D}}_{p}}{\partial HLB}\right)\right]}^{2},$$ (2)

where ${\hat{D}}_{p}$ is the predicted particle size. The penalty becomes active when the local model response violates the expected non-decreasing HLB–particle-size relationship.

#### 3.1.2 Hydration-temperature constraint

Hydration temperature affects surfactant mobility, bilayer fluidity, and vesicle self-assembly. The effect is not assumed to be universally monotonic because the response depends on the surfactant transition characteristics and formulation composition. Therefore, the temperature-related regularization is implemented as a **soft smoothness/consistency constraint** rather than as a fixed global positive or negative derivative. The corresponding penalty is represented generically as

$$P_{T}={\left[\mathrm{ReLU}\left(\left|\frac{\partial^{2}{\hat{D}}_{p}}{\partial T_{\mathrm{hyd}}^{2}}\right|-\tau_{T}\right)\right]}^{2},$$ (3)

where $\tau_{T}$ defines the maximum tolerated local curvature. This formulation discourages abrupt nonphysical oscillations while allowing formulation-dependent temperature responses.

#### 3.1.3 Cholesterol-to-surfactant constraint

Cholesterol modifies bilayer packing, membrane rigidity, permeability, and vesicle stability. Importantly, the direction of its effect on particle size is not universally identical across surfactant systems. Consequently, a fixed monotonicity assumption was not imposed. Instead, the regularization constrains excessive local sensitivity:

$$P_{C}={\left[\mathrm{ReLU}\left(\frac{\partial {\hat{D}}_{p}}{\partial (\mathrm{Chol}/\mathrm{Span})}-\tau_{C}\right)\right]}^{2}.$$ (4)

This allows the model to learn either an increasing or decreasing cholesterol effect when supported by the observations while discouraging unrealistically abrupt changes.

#### 3.1.4 Drug-to-lipid ratio constraint

The drug-to-lipid ratio affects drug loading, membrane organization, and vesicle structure. As with cholesterol, the response can depend on the physicochemical characteristics of the encapsulated drug and the formulation composition. The corresponding soft constraint is therefore expressed through a sensitivity penalty:

$$P_{D/L}={\left[\mathrm{ReLU}\left(\left|\frac{\partial {\hat{D}}_{p}}{\partial (D/L)}\right|-\tau_{D/L}\right)\right]}^{2}.$$ (5)

The complete physics-guided component is therefore

$$L_{\mathrm{physics}}=P_{\mathrm{HLB}}+P_{T}+P_{C}+P_{D/L}.$$ (6)

This formulation provides a reproducible description of the role of domain knowledge: HLB receives a directional consistency constraint, whereas variables with formulation-dependent responses are regulated through soft sensitivity constraints rather than forced into a universal monotonic relationship. The resulting regularization acts as an inductive bias rather than a hard physical law.

### 3.2 Generalization strategy

Cross-drug as well as cross-formulation generalization are aspects that are handled by a task-based learning approach where each task is a drug or a formulation family. This concept is inspired by the actual scenarios of drug development, where newly synthesized compounds are generally very limited in terms of particle size measurements. Through acquiring representations that can readily be adjusted by a small number of task-specific samples (40), the framework intends to lessen the dependency on dataset-specific biases and at the same time keep the capability of adapting to new systems.

### 3.3 Uncertainty-aware prediction

Uncertainty estimation is integrated into the predictive framework to provide risk-aware particle-size predictions. The learned latent representation generated by the neural feature encoder is passed to a probabilistic regression component based on a Gaussian-process uncertainty head. The probabilistic component produces a predictive mean and variance for each formulation (41). The predictive variance is decomposed into epistemic and aleatoric components:

$$\sigma_{\mathrm{pred}}^{2}=\sigma_{\mathrm{ep}}^{2}+\sigma_{\mathrm{al}}^{2}.$$ (7)

Here, $\sigma_{\mathrm{ep}}^{2}$ represents epistemic uncertainty, which reflects uncertainty associated with limited training information and imperfect knowledge of the latent prediction function. This component is expected to increase when the formulation lies in a sparsely represented region of the learned task space. The aleatoric component, $\sigma_{\mathrm{al}}^{2}$, represents irreducible observational uncertainty, including variability associated with experimental formulation and particle-size measurements. Unlike epistemic uncertainty, aleatoric uncertainty is not expected to disappear simply by increasing the amount of training data for the same experimental variability. For an input $x_{i}$, the probabilistic prediction is represented as

$$y_{i}∼N(\mu_{i},\sigma_{i}^{2}),$$ (8)

where $\mu_{i}$ is the predicted particle size and

$$\sigma_{i}^{2}=\sigma_{\mathrm{ep},i}^{2}+\sigma_{\mathrm{al},i}^{2}.$$ (9)

The negative log-likelihood used to assess the probabilistic prediction is

$$NLL=\frac{1}{N}\overset{N}{\underset{i=1}{\sum }}\left[\frac{{\left(y_{i}-\mu_{i}\right)}^{2}}{2\sigma_{i}^{2}}+\frac{1}{2}\log(2\pi \sigma_{i}^{2})\right].$$ (10)

The 95% prediction interval is obtained from the predictive distribution, and calibration is evaluated using PICP@95%. The uncertainty model was evaluated using the same independent train/test partition used for point prediction. The resulting decomposition is also examined at the individual-sample level. Increased epistemic uncertainty in samples with larger prediction deviations indicates reduced confidence in regions that are less represented by the available formulation data, whereas the comparatively stable aleatoric component reflects persistent experimental variability.

For comparative evaluation, a conventional ensemble baseline was also implemented. The baseline consisted of multiple independently trained predictive models generated using the same input-feature set and training partition as the proposed PGML framework. The ensemble point prediction was obtained by aggregating the predictions of the individual constituent models, whereas predictive uncertainty was estimated from the dispersion among the ensemble predictions. Thus, larger disagreement among individual ensemble members resulted in wider uncertainty estimates. The ensemble baseline did not include physics-guided regularization or task-based meta-learning and was therefore used to provide a conventional data-driven reference for evaluating the effect of the proposed integrated framework on point prediction and uncertainty behavior. All models were trained using the same preprocessing procedure and independent test partition to maintain a consistent comparison.

### 3.4 Training and optimization

Model training adopts a bi-level optimization framework to disentangle task-level adaptation from cross-task learning. The inner optimization loop executes the task-specific updates; at the same time, the outer loop implements the meta-level changes that aim at stable generalization across tasks [The inner loop uses the Adam optimizer (42),while the outer loop employs AdamW (43) for improved generalization.]. The regularization comprises L2 weight decay and physics-guided penalty terms, with penalty weights chosen by cross-validation. All tasks’ setups, data splits, and data preprocessing were done in such a way that information leakage was avoided, i.e., no validation or test data had any impact on training.

### 3.5 Performance evaluation metrics

Model performance was assessed by a suite of standard pointestimate and uncertainty-aware metrics that were purposely designed to mirror the objectives of predictive accuracy, explained variability, and uncertainty quantification. Firstly, RMSE and MAE determine the accuracy of predictions, R^2^ quantifies the amount of variance explained, PICP is used to check the uncertainty calibration, and NLL determines the overall quality of probabilistic prediction.

Predictive accuracy was measured by the RMSE and MAE. RMSE gives a greater weight to larger deviations and is very sensitive to huge prediction errors, whereas, MAE offers a more robust evaluation which is a good complement as it treats all errors equally. Equations 11–14 presents the evaluators formulas used to assess used model performance. These metrics are given by

$$\mathrm{RMSE}=\sqrt{\frac{1}{N}\overset{N}{\underset{i=1}{\sum }}{\left(y_{i}-{\hat{y}}_{i}\right)}^{2}},\;\mathrm{MAE}=\frac{1}{N}\overset{N}{\underset{i=1}{\sum }}|y_{i}-{\hat{y}}_{i}|,$$ (11)

where $y_{i}\;$ and ${\hat{y}}_{i}\;$ denote the observed and predicted values, respectively, and N is the number of samples.

The proportion of variance explained by the model was assessed using the R^2^, a normalized goodness-of-fit measure defined as

$$R^{2}=1-\frac{\sum_{i=1}^{N}{\left(y_{i}-{\hat{y}}_{i}\right)}^{2}}{\sum_{i=1}^{N}{\left(y_{i}-\bar{y}\right)}^{2}},$$ (12)

where $\bar{y}$ is the mean of the observed values.

Uncertainty calibration was evaluated using PICP@95%, which measures the proportion of observed values that fall within the corresponding predicted 95% prediction intervals. Therefore, PICP assesses empirical coverage relative to the nominal confidence level but does not quantify the width or sharpness of the prediction intervals. Interval magnitude was consequently considered separately when interpreting the concentration of predictive uncertainty. A model can achieve high empirical coverage by producing excessively wide intervals; therefore, coverage alone is insufficient for evaluating the practical usefulness of uncertainty estimates. PICP@95% is calculated as

$$\mathrm{PICP}=\frac{1}{N}\overset{N}{\underset{i=1}{\sum }}I[y_{i}\in [{\hat{y}}_{i}^{L},{\hat{y}}_{i}^{U}]],$$ (13)

where ${\hat{y}}_{i}^{L}$ and ${\hat{y}}_{i}^{U}$ denote the lower and upper bounds of the prediction interval, and I(⋅) is the indicator function.

Lastly, NLL was employed to measure the probabilistic predictive quality by combining both the mean error and the uncertainty calibration. The predictive distributions that are Gaussian, NLL is given as

$$\mathrm{NLL}=\frac{1}{N}\overset{N}{\underset{i=1}{\sum }}\left[\frac{\left(y_{i}{\hat{\mu }}_{i}^{2}\right)}{2{\hat{\sigma }}_{i}^{2}}+\frac{1}{2}\log(2\pi {\hat{\sigma }}_{i}^{2})\right],$$ (14)

where ${\hat{\mu }}_{i}$ and ${\hat{\sigma }}_{i}^{2}\;$ represent the predicted mean and variance, respectively.

Overall these commonly used metrics give a combined and easy-to-understand evaluation of how well the predictions are accurate, how much of the phenomena is correctly explained, and how well the predicted uncertainties are aligned with the observed ones, thus helping to evaluate the particle size prediction in a decision-support context without mixing up evaluation with interpretation of results.

## 4 Results and discussion

Figure 4 illustrates that the proposed physics-guided meta-learning model is trained in a numerically stable way under bi-level optimization. The meta-training loss shows a smooth and gradually decreasing pattern over 50 meta-epochs, without oscillation or divergence. Such a well-behaved meta-loss curve is really important especially with the small dataset size and heterogeneity of drug–formulation tasks that are the main reasons why MAML-type methods are unstable. The findings show that the physics-guided term addition serves as soft gradient guidance instead of a hard constraint, thereby enabling stable optimization during training. More importantly, validation RMSE, evaluated on held-out tasks, decreases from approximately 0.70 to 0.52 as meta-training progresses. This pattern indicates improving predictive performance across the held-out formulation tasks represented in the present dataset. Although the concurrent reduction in meta-training loss and validation RMSE is consistent with the learning of information that can be shared across the available tasks, it should not be interpreted as evidence of universally transferable formulation relationships. Given the limited dataset size and heterogeneity of the formulation families, additional validation using independent and broader formulation datasets is required to establish the extent of transferability beyond the experimental conditions represented in this study.

**Figure 4:**
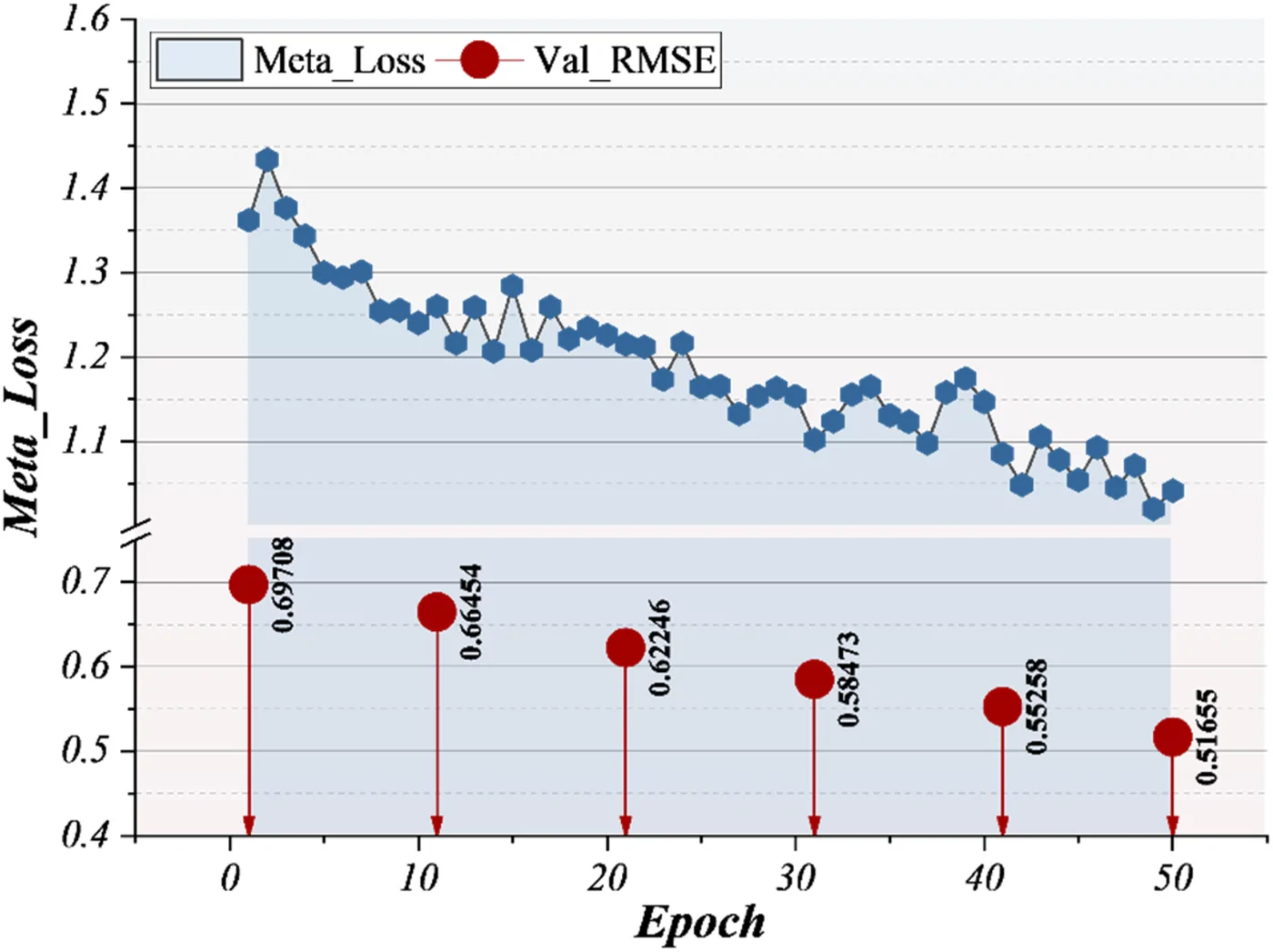
Meta-training convergence and generalization performance of the physics-guided meta-learning framework. The blue curve (with shaded area) shows the evolution of the meta-training loss (Meta_Loss) across epochs, reflecting stable optimization of the MAML backbone under physics-guided regularization. Red markers denote the validation root mean squared error (Val_RMSE) evaluated at selected meta-epochs, with annotated values highlighting progressive improvement in predictive accuracy across unseen drug–formulation tasks.

Figure 5 illustrates a joint assessment of predictive accuracy and uncertainty quality, where the proposed physics-guided meta-learning (PGML) framework is compared with an ensemble baseline. The PGML model achieves an RMSE of 0.6525, an MAE of 0.4323, and an R^2^ of 0.8935 on the independent test set. The ensemble baseline provides a slightly higher R^2^, but the difference in point-prediction performance is accompanied by substantially different uncertainty behavior. The PGML framework achieves PICP@95% of 0.9077, close to the nominal 95% level, while maintaining a substantially smaller average uncertainty of approximately 0.2324. In comparison, the ensemble produces substantially wider uncertainty estimates, resulting in an average uncertainty of approximately 4.07 × 10^8^ and an NLL of approximately 1.553. The PGML result therefore indicates a more compact and informative uncertainty representation while maintaining competitive point-prediction accuracy.

**Figure 5:**
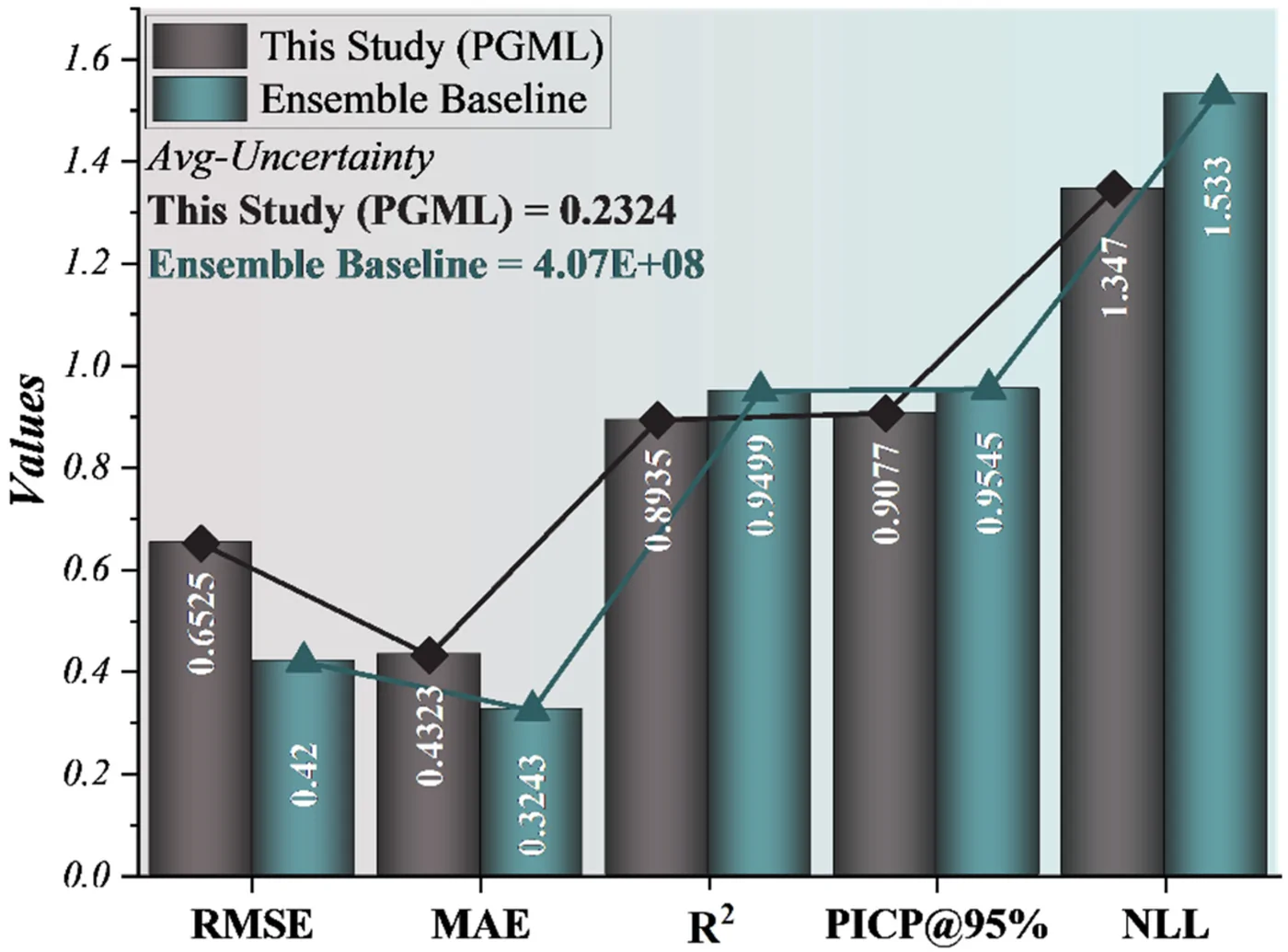
Comparison between the proposed physics-guided meta-learning (PGML) model and an ensemble baseline in terms of predictive performance and uncertainty.

Figure 6 shows sample-wise predictions of drug particle size using the proposed PGML framework on the fully independent test set, along with uncertainty decomposition results. Along with signed and absolute prediction errors, per each test sample, the true particle size value, predicted value, total predictive uncertainty, and its epistemic and aleatoric components are presented. Generally, the predicted particle size values e experimentally observed values for the most part of samples, while total uncertainty is moderate and stable, which means that the model is quite confident in its predictions in the regions well covered by the meta-training tasks. For some points, however, such as point 7 and points 16–18 where the prediction error seems to be high, these cases are accompanied by quite a significant increase in the epistemic uncertainty and, therefore, in the total uncertainty as well. Such a response shows that the model correctly conveys that it is less sure when dealing with drug–formulation tasks that are barely represented in the training dataset, as opposed to what the model does when it simply maintains the wrong level of the certainty. Meanwhile, the aleatoric uncertainty component varies only slightly from point to point and, thus, can be interpreted as the inherent random noise that is always present in particle size measurements, rather than limitations of the model knowledge. Furthermore, the agreement between high prediction errors and high epistemic uncertainty indicates that the PGML method provides well-calibrated uncertainty estimates that greatly facilitate trustworthy prediction interpretation and safety-aware decision making when screening for new formulations.

**Figure 6:**
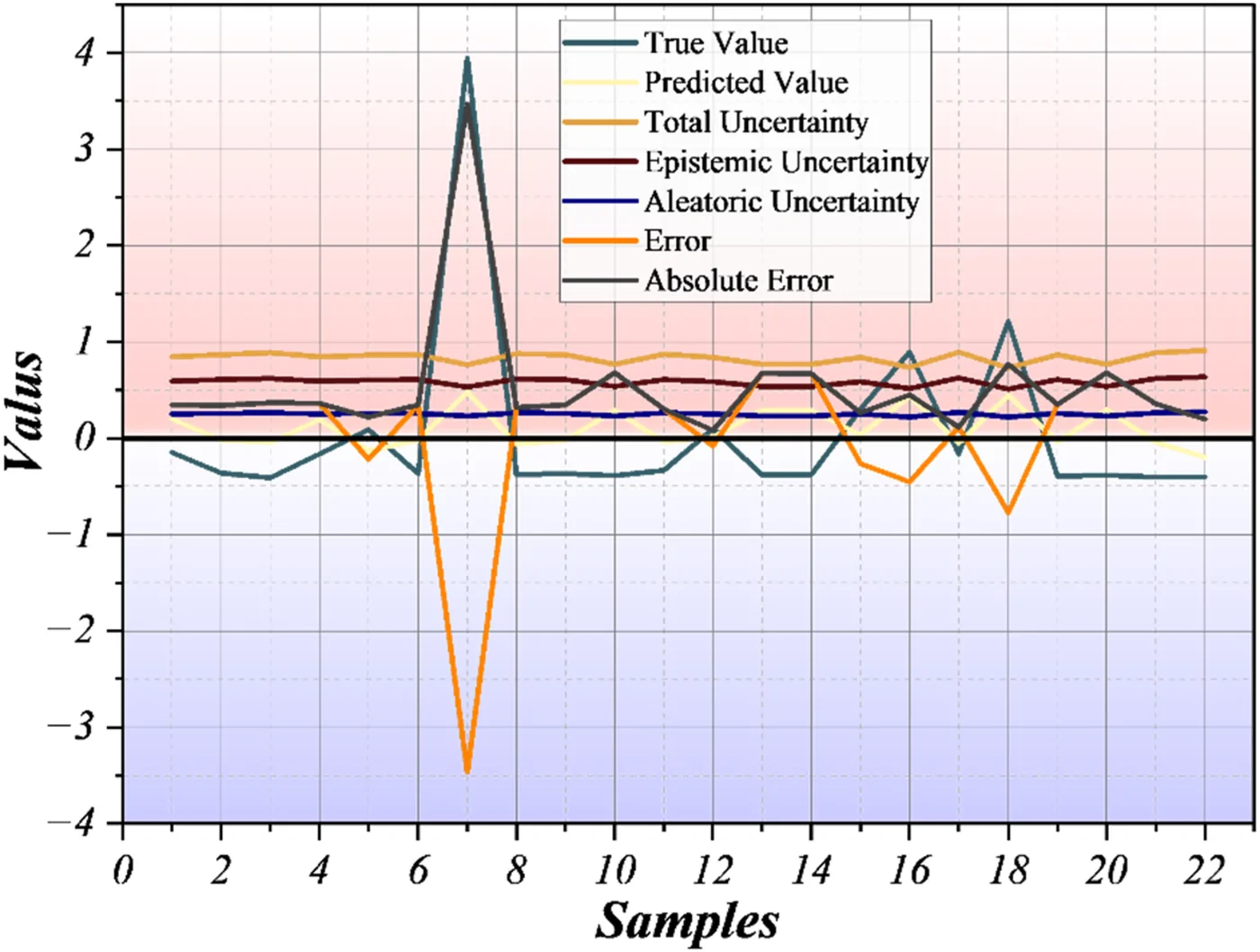
Sample-wise predictions of the PGML model on the test set showing true values, predicted values, total predictive uncertainty, and its decomposition into epistemic and aleatoric components, alongside prediction error. Increased epistemic uncertainty is observed in samples with larger prediction deviations, indicating self-aware and informative uncertainty behavior.

Figure 7 presents a summary of the final predictive performance and experimental configuration of the proposed PGML framework on the independent test set. The radar chart summarizes predictive accuracy through RMSE and MAE, goodness-of-fit through R^2^, and uncertainty calibration through PICP@95%. The final model achieves an RMSE of 0.6525, an MAE of 0.4323, an R^2^ of 0.8935, and a PICP@95% of 0.9077. The uncertainty coverage is therefore close to the nominal 95% level while maintaining a substantially lower uncertainty magnitude than the ensemble baseline. The accompanying configuration table reports 114 total observations, 92 training samples, 22 test samples, 11 input features, 50 meta-epochs, a physics weight of 0.2, an inner-loop learning rate of 0.001, a meta-level learning rate of 0.0001, and a 5-shot adaptation setting.

**Figure 7:**
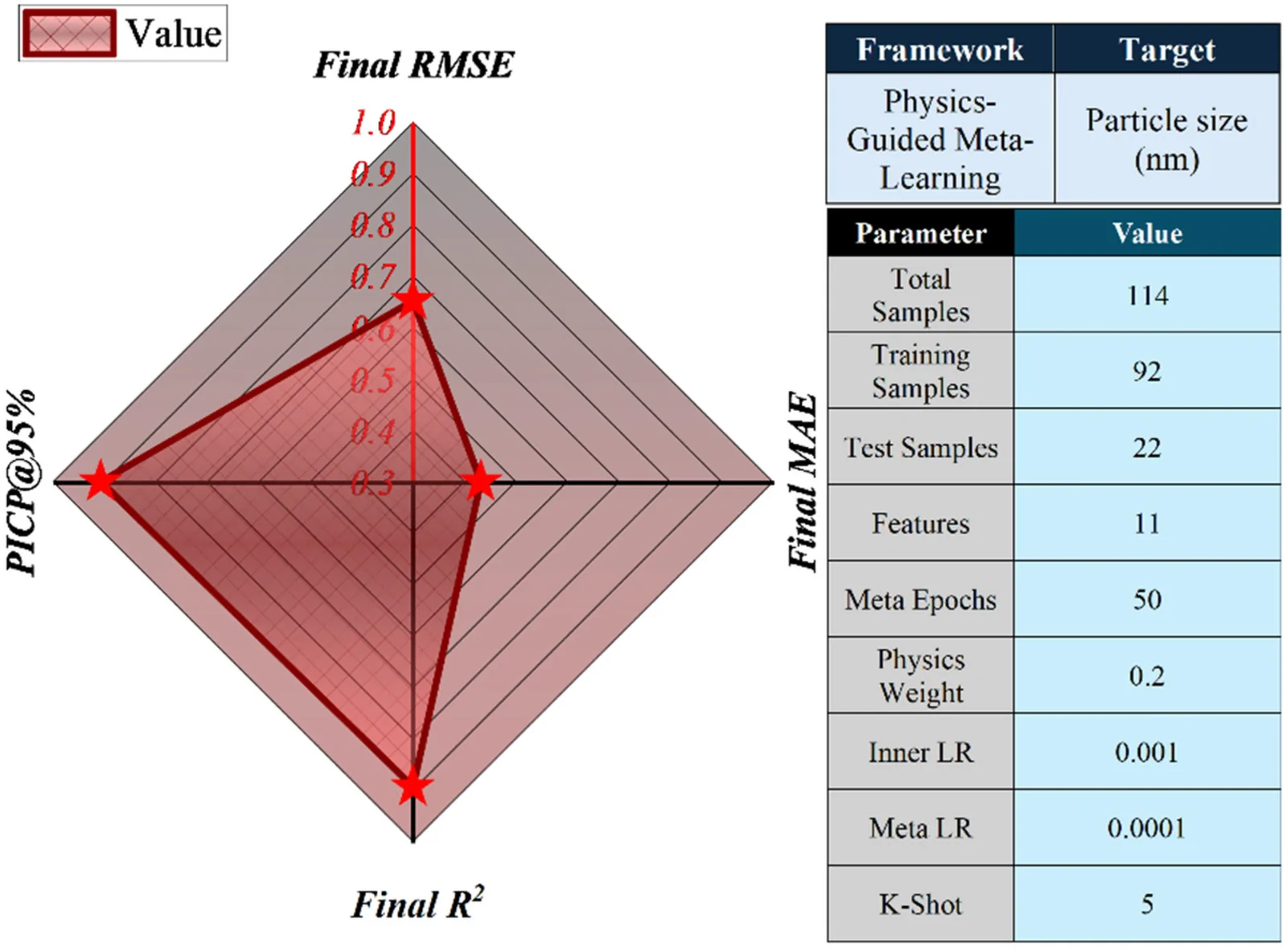
Summary of final PGML performance on the held-out test set. The radar chart aggregates RMSE, MAE, R^2^, and PICP@95%, illustrating balanced predictive accuracy and uncertainty coverage, while the accompanying table reports key dataset characteristics and meta-learning configuration parameters for reproducibility.

Figure 8 shows the mean absolute SHAP values for all input features and provides a global summary of their relative contributions to particle-size predictions within the PGML framework. To facilitate interpretation across different levels of system design, the features are categorized into molecular descriptors, formulation parameters, and processing conditions. Among the processing-related variables, hydration temperature and hydration time show the highest average contributions, indicating that the predicted particle size is strongly influenced by preparation conditions. The cholesterol-to-Span 60 ratio and the drug-to-lipid ratio also exhibit substantial contributions, which is consistent with their roles in vesicle structure, membrane rigidity, and drug encapsulation behavior. Physicochemical properties, including LogP, intrinsic aqueous solubility, pKa, and molecular weight, provide moderate but consistent contributions to the predictions. These results suggest that the model incorporates fundamental physicochemical characteristics of the drug while simultaneously accounting for the dominant influence of formulation composition and processing conditions. In contrast, HLB and solvent amount exhibit lower average SHAP values, indicating a comparatively smaller global contribution across the available dataset.

**Figure 8:**
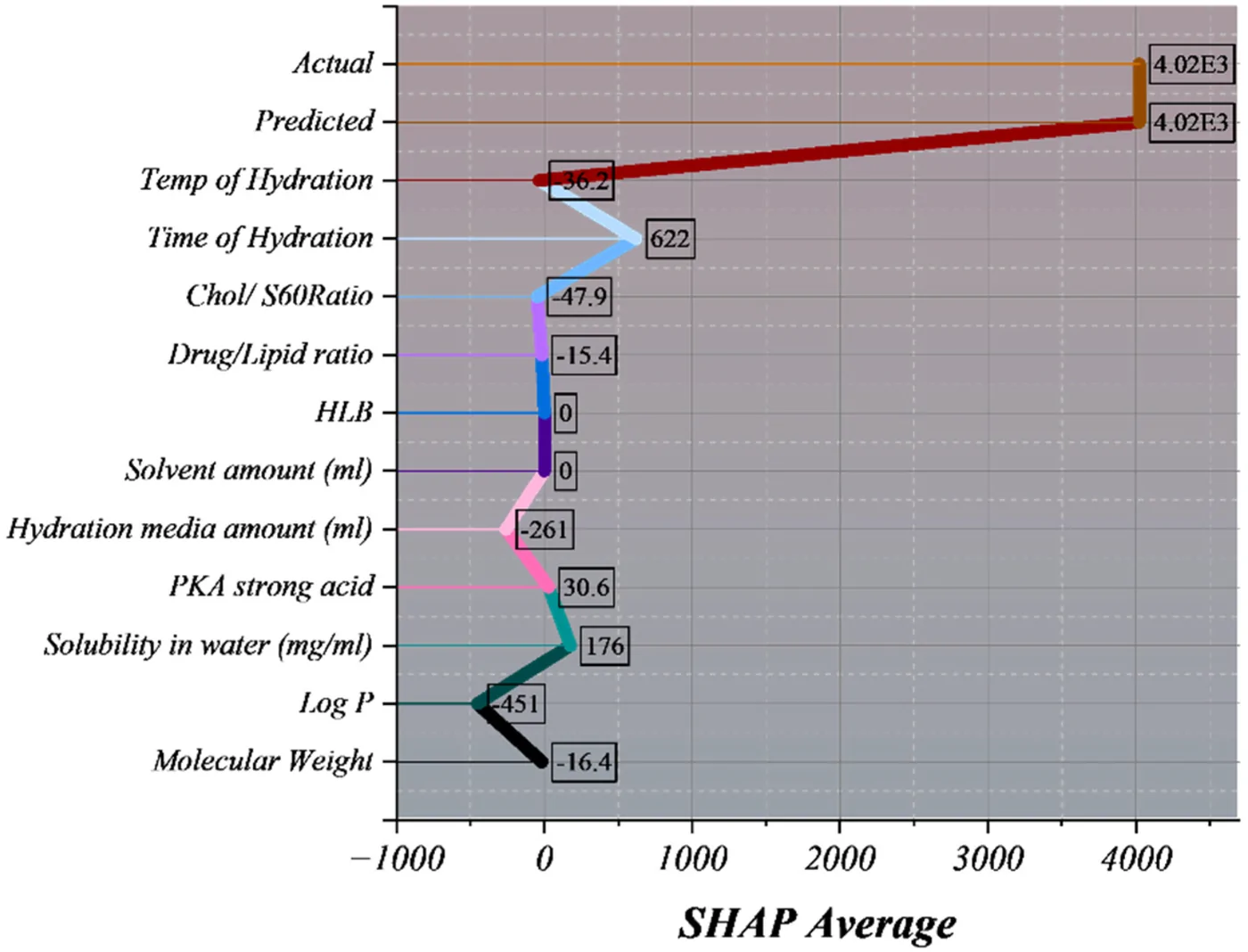
Global SHAP analysis showing average feature contributions to PGML particle size predictions. Processing conditions (hydration temperature and time) and key formulation ratios exhibit the strongest influence, while molecular descriptors provide consistent physicochemical context for model decisions.

### 4.1 Model behavior and practical relevance

Overall, the findings demonstrate how combining physics-guided regularization, meta-learning, and probabilistic uncertainty modeling not only impacts predictive capabilities but also influences how models are trained, how uncertainties are handled, and the level of explainability for prediction of particle size at the formulation level. Instead of pursuing the highest accuracy on point estimates, this framework is seen to align most closely with practical situations in formulations where data is scant, drug-formulation combinations are diverse, and trustworthy confidence indications are needed. Together, these results suggest that by physically consistent, task-generalizable, and uncertainty-aware methodologies are integrated within a single learning process, the resulting model behavior is not only stable but also capable of supporting real-world decision-making.

Through bi-level optimization, the stable meta-training dynamics attest that physics-guided regularization serves as a good inductive bias rather than a strict constraint. The meta-loss steadily diminishes during meta-training, and the validation results on held-out tasks keep getting better with no oscillations seen and RMSE values around ≈0.65. This is in stark contrast with the feature of instability commonly reported for MAML-based methods under task heterogeneity and low sample sizes. The pattern of convergence witnessed suggests the model has learned the essence of drug–formulation families that can be shared rather than locked in the specific task patterns, which is in line with the claim that physics guidance directs the optimization into areas of the parameter space that are in agreement with the physical laws.

A quantitative comparison with an ensemble baseline makes even more clear how the choices for structural modeling can influence the uncertainty behavior in a way that has a direct impact on decisions based on the experiment. On the one hand, both methods are able to produce very accurate point-predictions (given by R^2^ values around the range of ≈0.90–0.95), on the other hand, their probabilistic features are very different. The ensemble baseline manages to have coverage close to nominal (PICP@95% ≈ 0.95) mostly by making the confidence intervals very wide, at the same time, the uncertainty scale is extremely large (around the level of ≈10^8^) and the negative log-likelihood is high (NLL ≈ 1.5). The newly proposed framework, on the other side, manages to get the same level of coverage (PICP@95% ≈ 0.91) but the uncertainty level is very small, realistic (≈0.2–0.3), and the NLL is lower (≈1.35), which shows that the uncertainties are neither overestimated nor under consistent with the data.

Area case-wise decomposition of the uncertainties reveals still more features at the level of single predictions. High values of epistemic uncertainty are always accompanied by large prediction errors, while the distribution of errors for the samples with low epistemic uncertainty is quite narrow. However, in the meantime, the aleatoric factor does not change significantly from one sample to another, which is in line with the particle size experimental variability (random noise) rather than being due to a lack of model knowledge. The above quantitative disentanglement is very helpful for the risk-aware decision-making process as it makes it possible to separate the uncertainties due to limited prior knowledge from those that are due to the inevitable, irreducible experimental noise. From a purely operational point of view, the actions described are very typical of the early-stage development of new drug formulations. Keeping RMSE values under ≈0.7 while at the same time giving well-calibrated uncertainty is the key to efficient screening when particle size data are sparse, heterogeneous, and expensive to collect. The interpretability analysis also indicates that not only molecular descriptors but also processing variables like hydration temperature and time are making a chemically reasonable contribution. Essentially, this framework is meant to be a decision-support tool that, instead of being a purely predictive black box, allows uncertainty-aware exploration of formulation and processing spaces even under realistic data constraints and thus complements the experimental insights (Figure 9).

**Figure 9:**
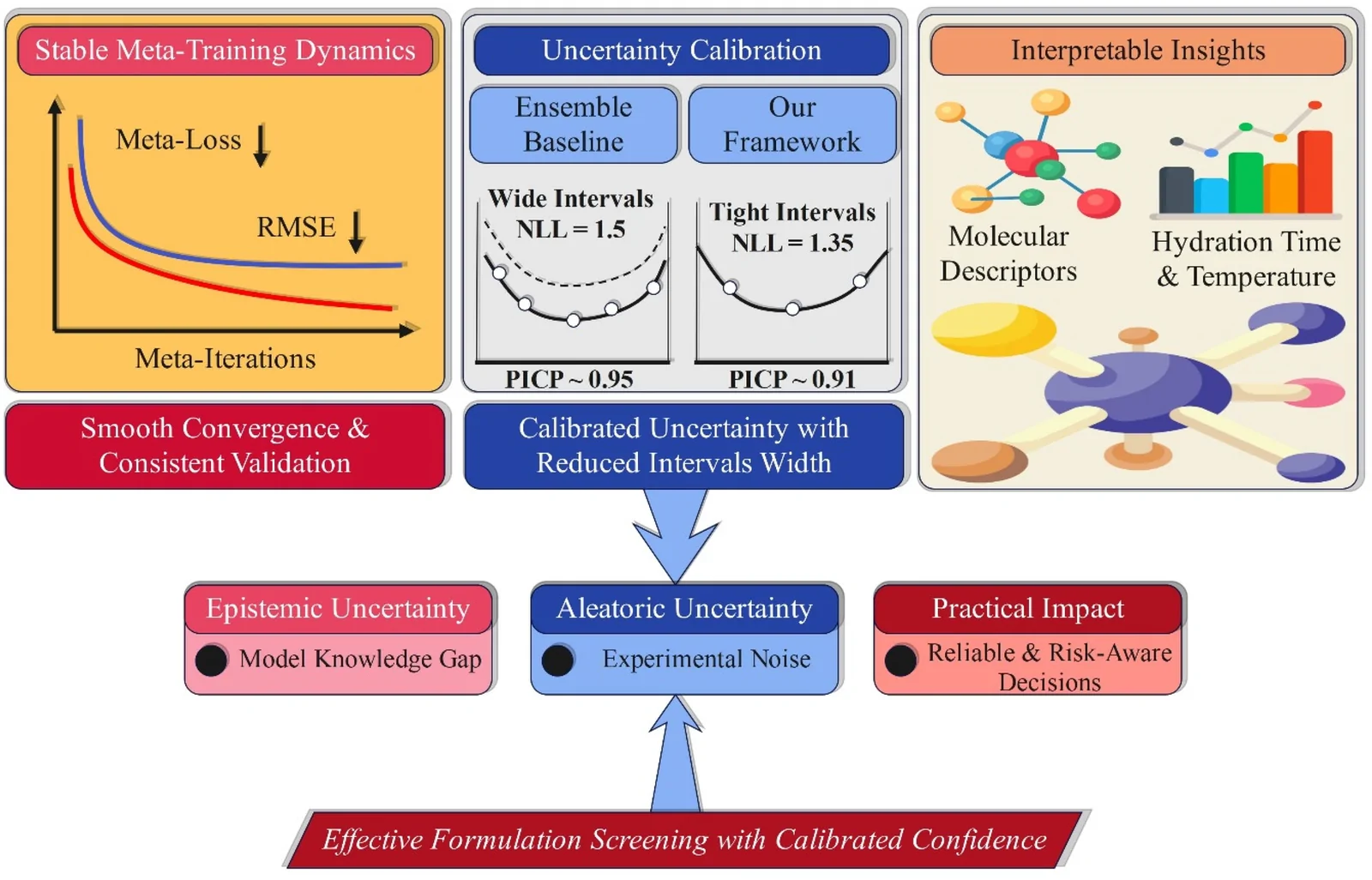
Conceptual overview of the physics-guided meta-learning framework, illustrating stable training dynamics, calibrated uncertainty decomposition, interpretable drivers, and their practical relevance for uncertainty-aware particle size prediction.

### 4.2 Limitation of the study

Several limitations should be considered when interpreting the present findings. First, the dataset contains 114 formulations collected from 17 independent published studies, which represents a relatively limited sample size for training and evaluating a complex meta-learning framework. Although the task-based design and independent test partition were intended to reduce overfitting and information leakage, the available data cannot fully represent the diversity of possible drugs, surfactants, formulation compositions, and manufacturing conditions encountered in pharmaceutical development. Second, the dataset was assembled from independent laboratories and published studies, and differences in experimental procedures, measurement instruments, reporting practices, and formulation protocols may introduce sources of heterogeneity that cannot be completely controlled during retrospective data curation. Such heterogeneity may affect both the observed particle-size values and the relationships learned by the model. Third, the present analysis is based primarily on experimental formulation and *in-vitro* characterization measurements and does not include clinical observations or patient-level outcomes. Consequently, the framework should be interpreted as a formulation-level decision-support approach rather than a direct predictor of clinical performance. Future studies should evaluate the proposed framework using larger and more standardized datasets, prospectively generated formulation experiments, and, where appropriate, additional biological or clinical endpoints.

## 5 Conclusion

The work presents an innovative integrated framework that combines physics-guided learning, meta-learning, and uncertainty quantification to predict mean particle size of formulations across a wide range of formulation and processing conditions. The study shows that by integrating physicochemical consistency with adaptation at the task level, it is possible to achieve stable generalization across different formulation systems. This even holds true when facing limited experimental data or data distributions. During meta-training period we saw that validation error reduced from approximately 0.70 down to 0.52 which was a very pleasing decrease. The proposed framework showed considerable alignment with experimental data (RMSE ≈ 0.65, R^2^ ≈ 0.90) on the held-out test set while the uncertainty remained around nominal level (PICP@95% ≈ 0.90). Besides, uncertainty was lower in the regions of the formulation space that contained many data points and it went up in the areas corresponding to the highest errors predicting that the model provides calibrated uncertainties which account for the two aspects namely limitations in knowledge (epistemic) as well as experimental variability. In-depth analysis of the model also pointed out the particle size is mainly affected by the composition of the formulation and the parameters used for processing where the contribution of molecular descriptors was in line with well-known physicochemical principles. The relationships revealed highlight the capability of the framework to model plausible experimental interactions among the formulation variables rather than just exploiting any superficial correlations. In total, the combination of physics-guided constraints that enforce domainknowledge, transferable meta-learning that facilitates sharing between different tasks, and uncertainty-aware prediction leads to a strong and interpretable method for particle size at formulation level prediction. The paper doesn't pretend the framework is a replacement for physical characterizing techniques. Still, it is expected that the framework would assist decision making in aspects like, for example, formulation screening, the prioritization of experiments and the optimization of the process, especially in cases when availability of experimental data and resources is limited.

## Data Availability

The original contributions presented in the study are included in the article/supplementary material, further inquiries can be directed to the corresponding author/s.
